# Potential sources of interference with the highly sensitive detection and quantification of alpha‐synuclein seeds by qRT‐QuIC

**DOI:** 10.1002/2211-5463.12844

**Published:** 2020-04-10

**Authors:** Viktoria C. Ruf, Song Shi, Felix Schmidt, Daniel Weckbecker, Georg S. Nübling, Uwe Ködel, Brit Mollenhauer, Armin Giese

**Affiliations:** ^1^ Center for Neuropathology and Prion Research Ludwig‐Maximilians‐University Munich Germany; ^2^ MODAG GmbH Wendelsheim Germany; ^3^ Department of Neurology University Hospital Munich Ludwig‐Maximilians‐University Munich Germany; ^4^ Paracelsus‐Elena‐Klinik Kassel Germany; ^5^ University Medical Center Göttingen Germany

**Keywords:** (q)RT‐QuIC, aggregation, alpha‐synuclein, biomarker, Parkinson’s disease, seeding

## Abstract

Parkinson’s disease (PD) is a progressive neurodegenerative disease which is histologically characterized by loss of dopaminergic neurons in the substantia nigra and deposition of aggregated alpha‐synuclein (aSyn) in the brain. The detection of aSyn in well accessible fluids has been one of the central approaches in the development of biomarkers for PD. Recently, real‐time quaking‐induced conversion (RT‐QuIC) has been successfully adapted for use with aSyn seeds. Here, we systematically analysed parameters potentially impacting the reliability of this assay by using quantitative real‐time quaking‐induced conversion (qRT‐QuIC) with *in vitro*‐formed aSyn seeds. Seeds diluted in cerebrospinal fluid (CSF) accelerated the seeding reaction and slightly increased the sensitivity without affecting specificity. Repeated freeze–thaw cycles decreased the apparent lag times of seeds diluted in ddH_2_O but did not alter the seeding activity of seeds diluted in CSF. High levels of artificial contamination with blood resulted in prolonged apparent lag times, while sensitivity and specificity were unaffected. Altogether, qRT‐QuIC with aSyn seems to be robust concerning sensitivity and specificity in our model system, but quantitative interpretation might be limited under certain conditions.

## Graphical abstract

RT‐QuIC with alpha‐synuclein represents a promising tool for the early diagnosis of synucleinopathies from cerebrospinal fluid. We tested typical conditions like repeated freeze–thaw cycles or contamination with blood which may impair the functionality of the assay. While freeze–thaw cycles do not seem to have a major impact, blood prolonged the apparent lag phases but did not change sensitivity or specificity of the assay.

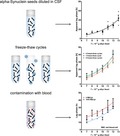

**Abbreviations**
(q)RT‐QuIC: (quantitative) real‐time quaking‐induced conversion
aSyn: alpha‐synuclein
CSF: cerebrospinal fluid
EM: electron microscopy
LP: lumbar puncture
NPH: normal pressure hydrocephalus
PD: Parkinson’s disease
RBC: red blood cells/red blood cell count
ThT: thioflavin T

Parkinson’s disease (PD) is a progressive neurodegenerative disease which is histologically characterized by loss of dopaminergic neurons in the substantia nigra and depositions of aggregated alpha‐synuclein (aSyn) in the brain. First clinical symptoms occur, when 50–70% of the nigral dopaminergic neurons are already lost [1, 2, 3], which is too late for effective and causative treatments. Thus, there is an urgent need for reliable biomarkers, allowing a sensitive and specific diagnosis in individual patients at an early, ideally pre‐symptomatic stage and enabling the monitoring of the disease progression as well as assessing the efficacy of therapeutic interventions. As aSyn plays a key role in PD pathogenesis, the detection of aSyn in easily accessible body fluids like blood, cerebrospinal fluid (CSF) or saliva has been one of the central approaches in the development of biomarkers for PD. Several studies have demonstrated that the levels of total aSyn in CSF of PD patients were significantly reduced compared to healthy controls [4, 5, 6]. However, as there exists a substantial overlap between PD patients and healthy controls on an individual basis, determining total aSyn in CSF as a single biomarker is presumably not sufficient to make a definite diagnosis. Attempts to detect pathological forms of aSyn such as phosphorylated aSyn [7] or aSyn oligomers [8, 9, 10] in CSF or blood have so far not been able to reliably distinguish healthy controls from patients either. Recently, real‐time quaking‐induced conversion (RT‐QuIC), also referred to as protein misfolding cyclic amplification by some authors and initially developed for the ultrasensitive detection of infectious prions [11, 12], has been successfully adapted for use with aSyn seeds and substrate and represents so far one of the most promising approaches [13, 14, 15, 16]. The underlying mechanism is based on the conversion of monomeric substrate protein into β‐sheet‐rich aggregates by seeding with minute amounts of protein aggregates and periodic shaking over several days. The amplification of protein aggregates is monitored in real‐time by an increase in thioflavin T (ThT) fluorescence. A further advancement of RT‐QuIC was established by our group with quantitative real‐time quaking‐induced conversion (qRT‐QuIC). Here, a standard calibration curve is calculated by quantitative correlation of distinct amounts of seeds and the apparent lag time, that is the time until the ThT fluorescence signal starts rising, which allows conclusions about the quantity of seeds applied and thus enables the monitoring of the disease progression and of therapeutic effects in prion‐infected mice [17, 18].

For the present study, we have used our qRT‐QuIC assay with *in vitro‐*formed aSyn seeds and substrate to systematically analyse parameters which in daily practice potentially interfere with the reliability of the assay.

## Materials and methods

### Expression and purification of recombinant aSyn substrate

Expression and purification were performed as previously described [19, 20]. Briefly, the pET5α/αSynuclein (136 TAT) plasmid (wt‐plasmid by Philipp Kahle, LMU Munich; 136‐TAC/TAT‐mutation by Matthias Habeck) was transformed into BL21(DE3) *Escherichia coli* (New England Biolabs, Frankfurt am Main, Germany). Protein expression was induced with 1 m IPTG (Peqlab, Erlangen, Germany) for 4 h at 37 °C. Cells were lysed by boiling after heat inactivation of proteases. After centrifugation, the supernatant was filtered through a Filtropur S 0.2 filter (Sarstedt, Nümbrecht, Germany), loaded onto a 5‐mL HiTrap Q HP anion exchange column (GE Healthcare, Munich, Germany) and eluted over a linear NaCl gradient (25–500 mm). The fractions containing aSyn were pooled, concentrated and gel filtrated via a Superdex 75 prep grade column (25 mL; GE Healthcare). After adjusting the protein concentration to 1 mg·mL^−1^, aliquots were frozen in liquid nitrogen and stored at −80 °C until use.

### Preparation of *in vitro*‐formed fibrils as seeds

Monomeric aSyn in 50 mm tris pH 7.0 was incubated at a concentration of 50 µm along with 100 mm NaCl and 0.02% NaN_3_ in a total volume of 1.4 mL at 37 °C for 96 h under vigorous orbital shaking (1400 r.p.m.) using an Eppendorf Thermomixer Comfort (Eppendorf, Hamburg, Germany) [21]. Fibril formation was confirmed by ThT fluorescence, electron microscopy (EM) and sucrose‐gradient centrifugation. After freezing in liquid nitrogen, fibrils were stored at −80 °C.

### ThT Fluorescence measurements

To measure the ThT fluorescence of *in vitro*‐formed fibrils, fibrils at a concentration of 5 µm were incubated with 50 µm ThT for 5 min at room temperature under constant stirring. ThT fluorescence spectra (460–560 nm) were subsequently recorded at an excitation wavelength of 450 nm using an LS 55 Luminescence Spectrometer (PerkinElmer, Waltham, MA, USA).

### Electron microscopy

Electron microscopy was carried out as previously described [21]. Briefly, undiluted fibril samples were applied onto carbon‐coated grids (Science Services, Munich, Germany) and treated with 1–2% uranyl acetate. Microscopy was performed using a Libra 120 transmission electron microscope (Carl Zeiss Microscopy GmbH, Oberkochen, Germany).

### Continuous sucrose‐gradient centrifugation

For sucrose‐gradient centrifugation, solutions containing 50 mm Tris (pH 7.4), 0.1% NP‐40 and sucrose (10%, 20%, 30%, 40%, 50% and 60%) were pipetted into a 4‐mL 11 × 60 mm polyallomer tube (Beckman Coulter, Brea, CA, USA) starting with 200 µL of 60% sucrose solution loaded to the bottom, followed by 400 µL layers of 50–10% sucrose solution. *In vitro‐*formed fibrils [200 µL at a concentration of 5 µm (monomer equivalent)] were loaded on the top of the sucrose gradient. Ultracentrifugation was performed with 40 000 r.p.m. (163 900 ***g***) at 4 °C for 1 h using a Sw60Ti rotor (Beckman Coulter). Samples were harvested after centrifugation from the top to the bottom of the sucrose gradient in 200‐µL fractions (i.e. fraction 1 represents the top of the gradient, fraction 12 the bottom fraction). 28 µL of each fraction was used for western blot analysis. aSyn was detected using an antibody against full‐length aSyn (4B12; BioLegend, San Diego, CA, USA).

### Lumbar puncture and CSF handling

All patients provided written informed consent to clinical assessment and lumbar puncture (LP) in order to collect CSF according to protocols approved by the local ethics committees (at University Medical Center Goettingen #36/7/02 and #9/7/04 and at LMU Munich #523 ‐ 16). All procedures of this study were in accordance with the 1964 Helsinki declaration and its later amendments or comparable ethical standards. Diagnostic/therapeutic high‐volume LP was performed in patients with normal pressure hydrocephalus (NPH). After the first 2–3 drops were discarded, 30–40 mL CSF was withdrawn and collected into 15‐mL polypropylene tubes. A sample was sent for routine analysis (cell count, total protein, glucose etc.). The remaining CSF was centrifuged at 2500 ***g*** for 10 min at room temperature, subsequently transferred to new tubes and frozen at −80 °C within 30 min.

### qRT‐QuIC

The qRT‐QuIC protocol is based on the protocol previously described by Shi *et al*. [17]: for a final reaction volume of 100 µL, 0.1 mg·mL^−1^ monomeric aSyn and 10 µm ThT were pipetted into a black 96‐well plate (Thermo Scientific, Waltham, MA, USA) in the presence of 1× QuIC buffer (10× QuIC buffer: 200 mm NaPi, 10 mm EDTA, 1.3 M NaCl pH 6.9). Seeds were diluted in ddH_2_O or CSF in 0.5‐mL Protein LoBind Tubes (Eppendorf AG) and added at final concentrations of 500 nm–500 fm (monomer equivalent), corresponding to 7 × 10^−7^–7 × 10^−13^ g of aSyn seeds, to the seeding reaction. The plate was immediately sealed with transparent tape (Kisker Biotech, Steinfurt, Deutschland) in order to avoid cross‐contamination and aerosol formation. Reactions were performed on FLUOstar OPTIMA and FLUOStar Omega multiwell plate readers (BMG Labtech, Ortenberg, Germany) at 50 °C for at least 90 h with cycles of 1 min double‐orbital shaking at 600 r.p.m. followed by 1 min of stationary incubation. ThT fluorescence was recorded every hour using an excitation wavelength of 440 nm and an emission wavelength of 480 nm. Apparent lag times were determined in a blinded manner and defined by the last time point before the ThT signal increased compared to the baseline. At least three independent experiments with duplicates or triplicates per seed concentration were performed for all different conditions.

For repeated freeze–thaw cycles, three dilution series (each 500 nm–5 pm) of seeds in ddH_2_O or CSF were prepared at a time and 0, 1 or 5 times frozen in liquid nitrogen and subsequently thawed at room temperature. Of note, always the whole dilution series with all subsequent dilutions were subjected to the number of freeze–thaw cycles indicated.

To mimic contamination with blood, blood by courtesy of two healthy volunteers [volunteer 1: sex: F, age: 30–35, red blood cell count (RBC): 4.6 × 10^6^ per µL; volunteer 2: sex: M, age: 30–35, RBC: 5.2 × 10^6^ per µL], of whom verbal informed consent was obtained, was collected into unlabelled EDTA‐coated test tubes (EDTA K^3^ S‐Monovette Hämatologie 2.6 mL; Sarstedt). Subsequently, the whole blood sample was diluted in CSF to the number of RBC indicated and the prepared CSF samples were spiked with aSyn seeds as specified above. All procedures of this study were in accordance with the 1964 Helsinki declaration and its later amendments or comparable ethical standards.

## Results

### Characterization of *in vitro*‐formed seeds

**Fig. 1:**
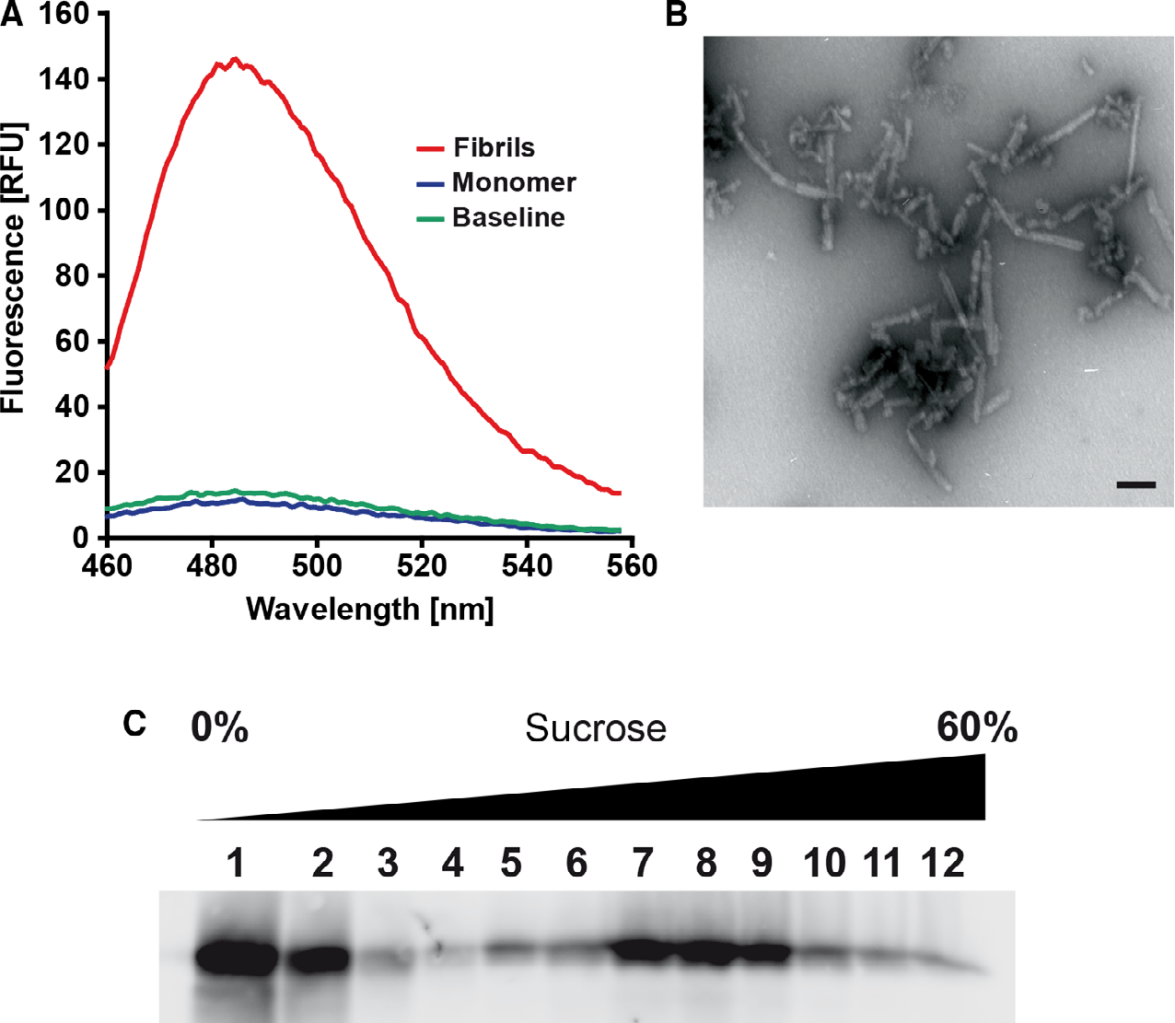
Characterization of *in vitro‐*formed seeds. (A) Compared to monomeric aSyn or buffer (baseline), the aSyn seed preparation shows a strong ThT fluorescence signal indicating formation of amyloid fibrils which was (B) further confirmed by EM; scale bar 100 nm. (C) Sucrose‐gradient centrifugation depicts a continuous distribution of differently sized aSyn aggregates over all fractions with a peak in fractions 7–9.

To adapt qRT‐QuIC for use with aSyn seeds and to optimize the assay conditions, we used artificial, *in vitro*‐aggregated aSyn as seeds to provide standardized conditions with low interexperimental variation and to save precious and irretrievable patient samples. aSyn seeds were prepared by incubating monomeric aSyn at a concentration of 50 µm for 96 h at 37 °C under vigorous orbital shaking at 1400 r.p.m. ThT fluorescence measurements of the seed preparations obtained showed an increased fluorescence intensity compared to buffer (baseline) or aSyn monomer (Fig. 1A), indicating that the *in vitro*‐formed seed preparations consist at least in part of amyloid fibrils which could be confirmed by EM (Fig. 1B). To retrieve more information about the size of the aggregates present, we performed sucrose‐gradient centrifugation, where we observed a continuous distribution of aSyn aggregates of different sizes peaking in fractions 7–9 (Fig. 1C).

### Adaptation of qRT‐QuIC for use with aSyn seeds and substrate

**Fig. 2:**
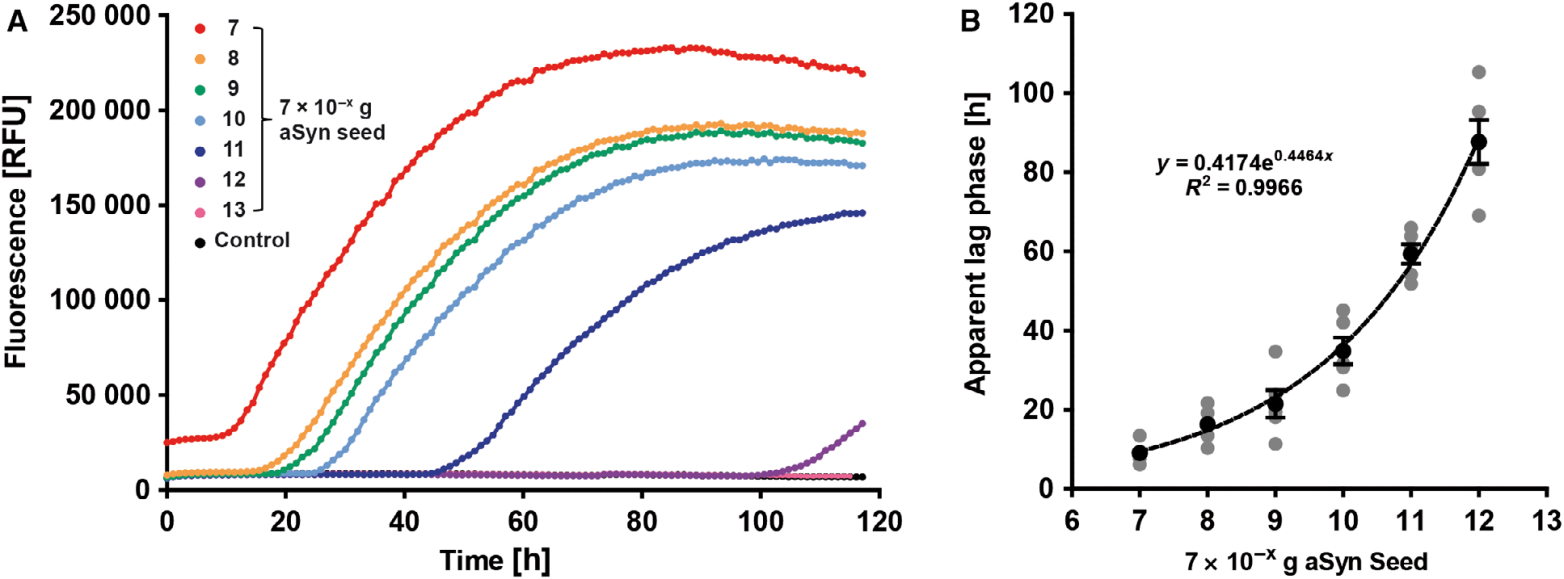
Adapting qRT‐QuIC for aSyn seeds. (A) Monomeric aSyn at a concentration of 0.1 mg·mL^−1^ was used as substrate and decreasing amounts of *in vitro‐*formed seeds were added to the reaction. A reliable ThT fluorescence signal was detected for seed amounts as low as 7 × 10^−12^ g. For 7 × 10^−13^ g of aSyn seeds, no signal could be detected by qRT‐QuIC anymore. (B) With decreasing amounts of seeds, the apparent lag times increased in an exponential manner. Each single grey dot represents the mean of one independent experiment (*n* = 4), and black dots represent the mean of the four independent experiments; error bars indicate SEM.

Based on the protocol described by Shi *et al*. [17], which had been optimized for the ultrasensitive detection of minute amounts of pathological prion protein, we adapted the assay conditions for the use with aSyn seeds. We found that a substrate concentration of 0.1 mg·mL^−1^ was ideal to reliably detect amounts of aSyn seeds as low as 7 × 10^−12^ g (monomer equivalent) (Fig. 2A) without self‐aggregation of the substrate within the observation time. Increasing the reaction temperature from 37 to 50 °C strongly accelerated the seeding reaction (Fig. S1). Shaking conditions were maintained according to Shi *et al*. [17]: 1 min double‐orbital shaking at 600 r.p.m. followed by 1 min of stationary incubation with recording of ThT fluorescence every hour. Quantitative analysis of the apparent lag times revealed an exponential correlation between the amount of seeds applied and the apparent lag time (Fig. 2B).

To see if and to what extent particular pre‐treatments of the seed preparations might affect their seeding activity, single experiments were performed where the seed preparation was ultracentrifuged with 47 000 r.p.m. (135 800 ***g***) at 20 °C for 30 min to possibly increase the concentration of active seeds or where the seed preparation was sonicated to receive a more homogeneous seed population (Fig. S2). However, as there were no obvious differences in the seeding activity after ultracentrifugation or sonication compared to conditions without pre‐treatment, we decided to use the seed preparations without further processing.

### CSF matrix does not inhibit but slightly increases the rate of aggregation induced by artificial seeds

**Fig. 3:**
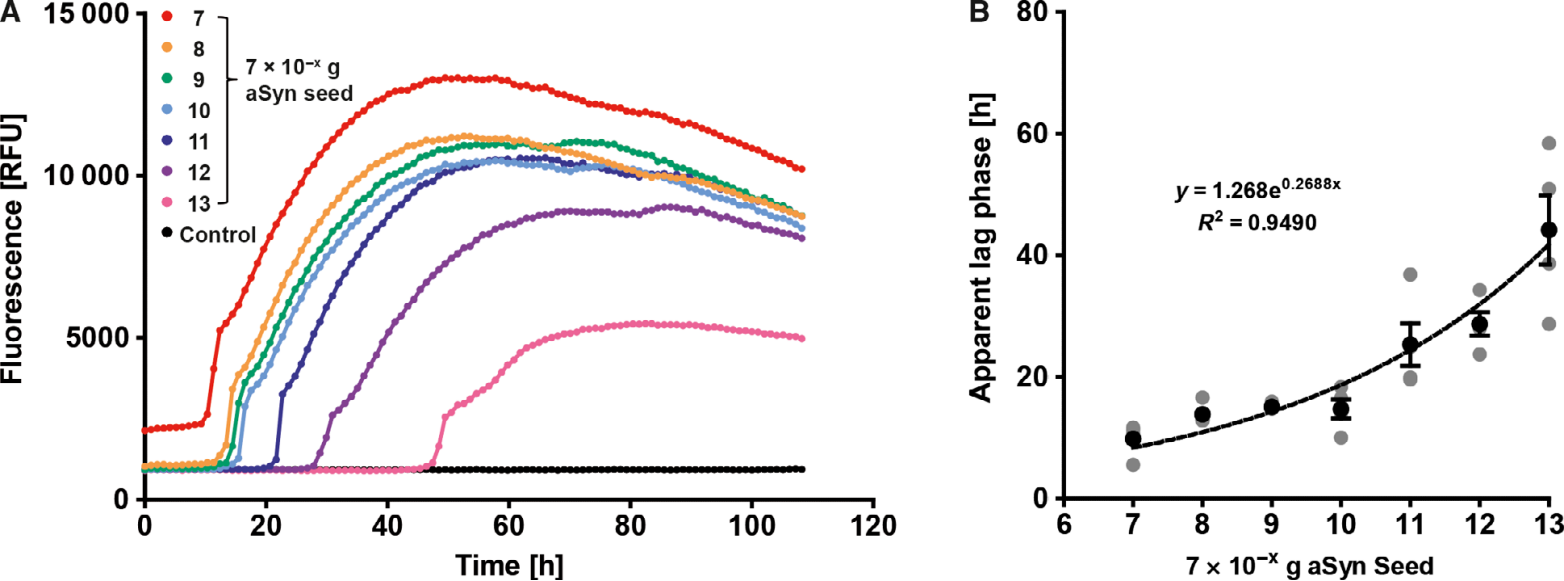
CSF matrix does not inhibit aggregation of *in vitro‐*formed seeds. (A) With aSyn seeds spiked in CSF, the apparent lag times were slightly shorter compared to the same amounts of aSyn seeds diluted in ddH_2_O. However, the aggregation *per se* was not impaired and showed as well an exponential association between the amount of applied seeds and apparent lag time (B). Each single grey dot represents the mean of one independent experiment (*n* = 4), and black dots represent the mean of the four independent experiments; error bars indicate SEM.

Cerebrospinal fluid consists of a variety of different components such as proteins, especially albumin, electrolytes, glucose and small amounts of cells. All these factors potentially interfere with the seeding reaction. As it is very difficult to artificially mimic the complex composition of CSF, we spiked CSF from patients with NPH, from which high volumes of CSF were obtained, with *in vitro*‐formed aSyn seeds and performed qRT‐QuIC to analyse, in what way the CSF matrix would affect the aggregation process. When seeds were diluted in CSF, we found a marked decrease of the apparent lag times compared to seeds diluted in ddH_2_O (Fig. 3A), while there was still an exponential correlation between the amount of seeds and lag times (Fig. 3B). Moreover, we reliably observed a seeding reaction with 7 × 10^−13 ^g aSyn seeds, indicating a slightly increased sensitivity of the assay with seeds diluted in CSF. However, for reasons of practicability, all subsequent experiments were performed with 7 × 10^−12^ g aSyn seed as the lowest amount of seeds.

### Freeze–thaw cycles do not affect the seeding activity of seeds diluted in CSF but reduce the apparent lag time of seeds diluted in ddH_2_O

**Fig. 4:**
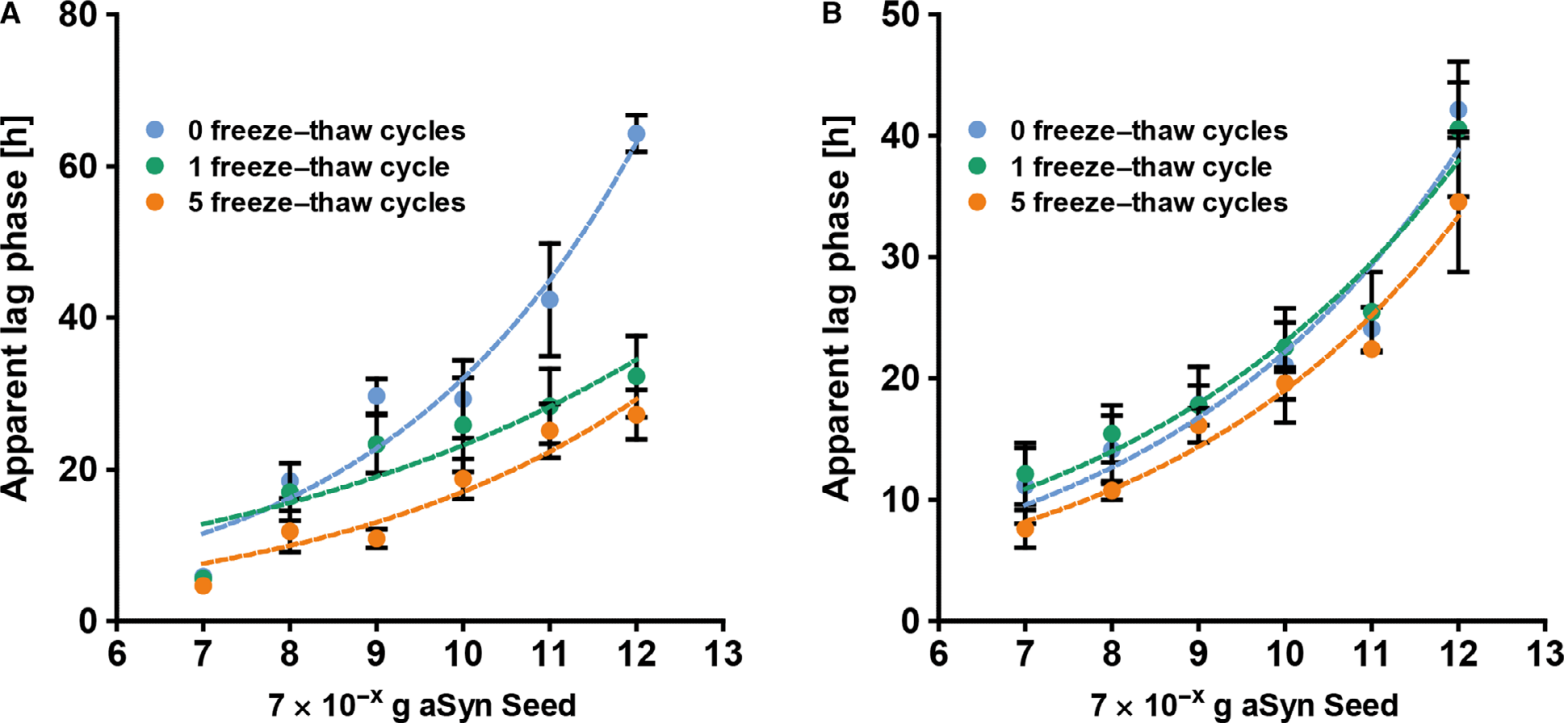
Freeze–thaw cycles accelerate the seeding reaction when seeds are diluted in ddH_2_O but have no effect on seeds diluted in CSF. Seeds diluted in either ddH_2_O or CSF at the concentrations indicated were subjected to 0, 1 or 5 freeze–thaw cycles immediately prior to being applied for the experiment. While a clear difference in apparent lag times was detected for seeds diluted in ddH_2_O (A), no obvious difference could be observed after 1 or 5 freeze–thaw cycles when CSF was spiked with aSyn seeds (B). Single dots represent the mean of *n* = 3 independent experiments; error bars indicate SEM.

In clinical routine, analyses of patient samples are usually performed in a sequential, stepwise process; thus, patient samples may undergo several freeze–thaw cycles until a particular test is performed. As this might have an impact on the seeding properties of the seeds in CSF samples, we tried to assess the seeding activity of *in vitro‐*formed seeds after one and five freeze–thaw cycles compared to seeds without freezing and thawing. When seeds were diluted in ddH_2_O, a strong decrease of the lag times especially at lower seed concentrations could be observed with only one freeze–thaw cycle. After five freeze–thaw cycles, this effect was even more pronounced (Fig. 4A). However, for seeds diluted in CSF, no differences between seeds with and without freeze–thaw cycles could be detected (Fig. 4B).

### Contamination with blood does not interfere with sensitivity or specificity of qRT‐QuIC but leads to increased apparent lag times

**Fig. 5:**
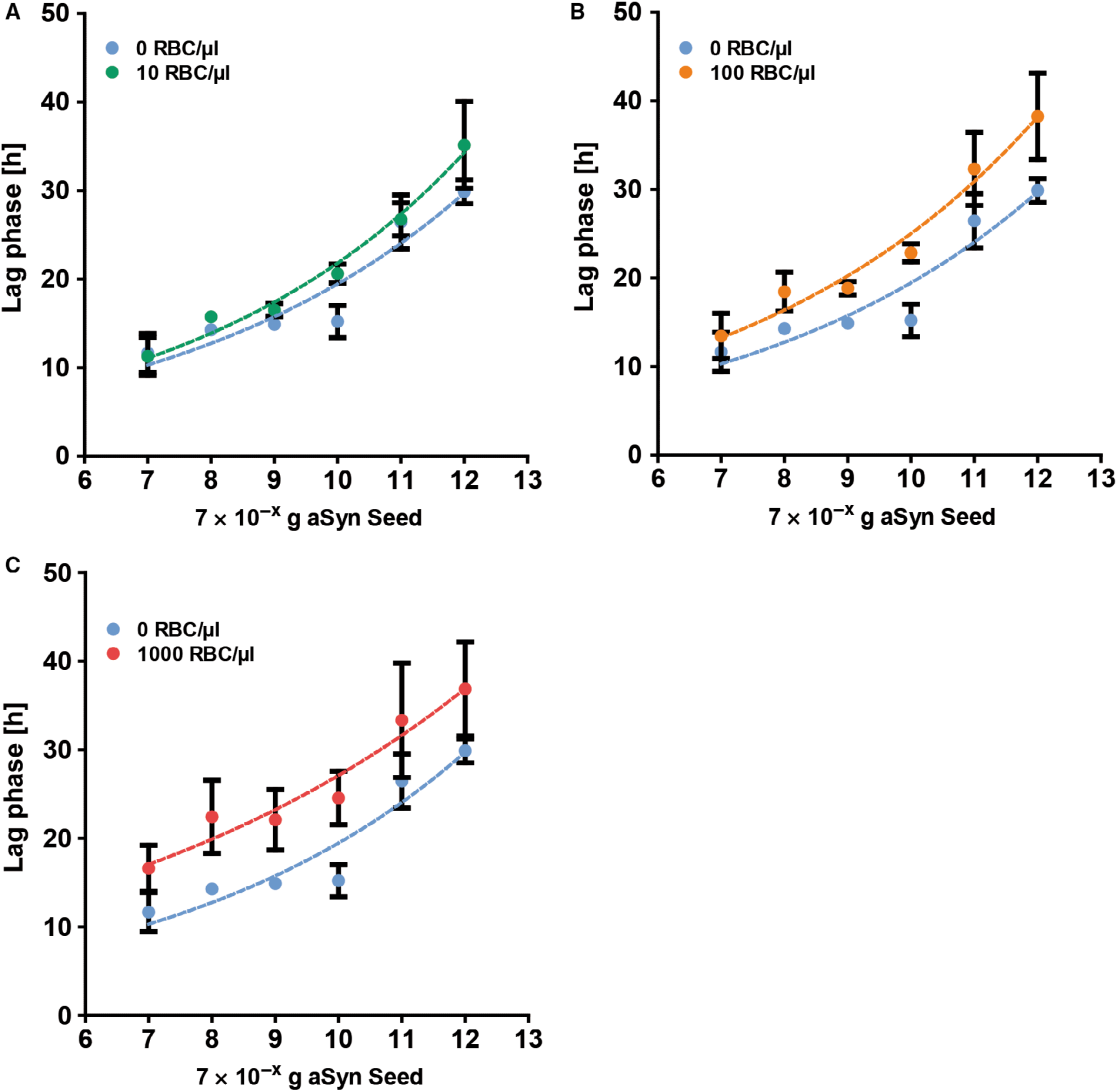
Contamination with blood prolongs apparent lag times. To mimic contamination with blood, whole blood of healthy volunteers was diluted to an estimated RBC of 1000, 100 and 10 RBC per µL, respectively, in CSF, which was spiked with aSyn seeds at the amounts indicated. (A) No obvious differences regarding apparent lag times could be detected with 10 RBC per µL. (B) With 100 RBC per µL, apparent lag times slightly and (C) with 1000 RBC per µL clearly increased in a comparable manner for all amounts of aSyn seeds tested. Each single dot represents the mean of *n* = 4 independent experiments; error bars indicate SEM.

A common problem for the analysis of CSF samples is contamination with blood either during LP or due to previous bleeding into CSF spaces, for example following craniocerebral injury after falling. Thus, we sought to investigate to what extent contamination with blood might interfere with the seeding reaction in a qualitative and quantitative manner. Therefore, whole blood from healthy volunteers (assuming an average RBC count of 5 000 000 RBC per µL) was diluted to final concentrations of 1000, 100 and 10 RBC per µL in the CSF sample to be analysed. While with 1000 RBC per µL a discrete reddish discolouration was still visible, admixtures of 100 or 10 RBC per µL could not be detected by eye anymore. Increasing blood concentrations led to a nearly parallel increase of the apparent lag times for all amounts of seeds tested, with an average increase of 2.3 h at 10 RBC per µL (Fig. 5A), 5.3 h at 100 RBC per µL (Fig. 5B) and 7.2 h at 1000 RBC per µL compared to 0 RBC per µL (Fig. 5C). Regarding sensitivity and specificity, no differences could be observed between different blood concentrations: Amounts of seeds as low as 7 × 10^−12^ g were able to induce a significant increase in ThT fluorescence, while the rate of false‐positive reactions was not increased in samples with artificial contamination with blood.

## Discussion

Neurodegenerative diseases like Alzheimer’s and Parkinson's disease have become a growing problem in our ageing population, as no causal and only limited symptomatic therapy is available to date. To provide effective and causative treatment strategies, an early and reliable diagnosis of specific neurodegenerative disease entities is required. Various approaches to develop sensitive and specific biomarkers to diagnose synucleinopathies or other neurodegenerative diseases have been followed, of which qRT‐QuIC seems currently to be one of the most promising approaches. We and others have adapted qRT‐QuIC for the use with aSyn seeds and substrate. In this study, we have systematically characterized factors of daily routine, which potentially interfere with the reliability of the assay.

To adapt qRT‐QuIC for use with aSyn seeds and substrate, we used *in vitro‐*formed aSyn seeds, which we characterized by ThT fluorescence measurements, continuous sucrose‐gradient centrifugation, and EM. ThT fluorescence measurements revealed strong fluorescence intensities for the seed preparations compared to buffer or monomeric protein, indicating the presence of amyloid fibrils, which could be confirmed by EM. Using sucrose‐gradient centrifugation, we could demonstrate a continuous size distribution suggesting that the seed preparation contains a variety of different‐sized aggregates. To address the question, if it was possible to enrich the fraction of active seeds or to retrieve a more homogenous seed population, single experiments were performed, where the seed preparation was ultracentrifuged or sonicated. However, we could not find obvious differences of the seeding activity compared to unprocessed seed preparations. Moreover, as it is unclear which particular aggregate species in fact trigger the seeding reaction and as there possibly exists a spectrum of different aggregate species in patient brains and CSF, we decided to proceed without further processing of the seed preparations.

To adapt qRT‐QuIC for use with aSyn seeds and substrate, we diluted the seed preparations by factors of ten in ddH_2_O, thus, reaching absolute amounts of 7 × 10^−7^–7 × 10^−13^ g of aSyn seeds, corresponding to concentrations of 500 nm–500 fm of aSyn seeds in the final reaction. A reaction temperature of 50 °C was chosen, as the seeding reaction seems to be reasonably fast. Moreover, at this temperature, microbial growth is virtually avoided, while at the same time there is no risk of protein denaturation yet. However, homogeneous heating of the multiwell plate should be ensured, and plates should be densely sealed to prevent evaporation. Decreasing seed concentrations led to increasing apparent lag times, which are correlated by an exponential relationship. This was used to establish a standard calibration curve.

As CSF is a body fluid of complex composition, we wanted to ensure that the CSF matrix *per se* does not impair the seeding reaction. Therefore, we spiked CSF with *in vitro*‐formed seeds and performed qRT‐QuIC under the same conditions as described before. In contrast to seeds diluted in ddH_2_O, we reliably observed seeding activity at 7 × 10^−13^ g aSyn seed. The lag times were generally shorter, especially at lower seed concentrations. Specificity was not affected. A possible explanation for the more sensitive and faster reaction could be that the functional seed concentration is increased, as the additional proteins from CSF might prevent the adsorption of seeds to the walls of the wells. Assuming a normal CSF protein concentration of 1.5–4.5 mg·mL^−1^, the concentration of protein in the reaction well would be at least doubled compared to our experiments with seeds diluted in ddH_2_O, where the aSyn substrate displays the only considerable protein source.

As clinical diagnostics is typically a sequential, multi‐step process, it is inevitable to store samples refrigerated or frozen over certain time periods and thaw them again for subsequent analyses. Thus, we tested the seeding activity of the *in vitro*‐formed seeds after one and five freeze–thaw cycles. While there was no obvious difference for CSF spiked with seeds, already one freeze–thaw cycle of seeds diluted in ddH_2_O was associated with a remarkable reduction of the apparent lag times, especially at lower seed concentrations. A potential explanation for this could be an increased fractionation of seeds diluted in ddH_2_O leading to a higher functional concentration of seeds (while the protein concentration remains the same) and thus to a reduction of the apparent lag phases. In contrast, seeds diluted in CSF might be protected against fragmentation due to the composition of CSF so that the functional concentration of seeds would not change. This hypothesis is supported by the observation that the ThT fluorescence of seeds diluted in ddH_2_O is clearly reduced after one or five freeze–thaw cycles, whereas only a slight reduction of the ThT fluorescence was recorded for seeds diluted in CSF (Fig. S3). In addition, it might be possible that seeds diluted in ddH_2_O undergo structural alterations during freeze–thaw cycles resulting in a different seeding activity. As we have observed changes in the apparent lag phases only with seeds diluted in ddH_2_O but not with seeds diluted in CSF, we assume that repeated freezing and thawing of CSF samples will most likely not substantially affect the activity of the seeds present in CSF. Therefore, we did not further follow up our observations on seeds diluted in ddH_2_O. Contamination of CSF samples with blood is commonly observed in daily routine and might interfere with the seeding reaction leading to erroneous results. To analyse the impact of contamination with blood, we mimicked contamination with blood by diluting whole blood of two healthy volunteers in CSF to estimated concentrations of 1000, 100 and 10 RBC per µL, respectively. While at 1000 RBC per µL, a discretely reddish discolouring of the CSF sample could still be observed by eye, CSF samples with 100 or 10 RBC per µL showed no visible discolouring. Increasing RBC counts caused a similar prolongation of the apparent lag times for all amounts of seeds tested leading to a nearly parallel shift of the calibration curve towards prolonged lag times. While the average delay was with 2.3 h comparably small for 10 vs 0 RBC per µL, lager differences of 5.3 and 7.2 h were detected for 100 and 1000 RBC per µL, respectively, so that especially the delay of the lag times at 1000 RBC per µL would lead to a considerable underestimation of the amount of seeds. However, sensitivity and specificity of the assay were not affected by any extent of artificial contamination with blood. About the reasons for the prolonged lag times we can only speculate: as it is well known that blood and particularly erythrocytes [22, 23, 24] contain high levels of aSyn, blood‐derived aSyn might have a relevant impact on the aggregation assay. Assuming a concentration of around 25 000 ng·mL^−1^ aSyn in blood [23], the maximum concentration of blood‐derived aSyn in our assay would be 0.5 ng·mL^−1^ and, thus, negligible given the substrate concentration of 0.1 mg·mL^−1^ aSyn. Therefore, it seems unlikely that blood‐derived aSyn has a significant impact on the aggregation reaction. Moreover, one would probably expect a decrease in lag times with higher amounts of aSyn in contrast to the observed increase. Another factor that needs to be considered is serum albumin, the most abundant protein in blood and known to inhibit the aggregation of various amyloidogenic proteins such as aSyn [25, 26, 27]. However, with an average albumin concentration of 3.5–5 g·L^−1^ in blood, the maximum concentration of blood‐derived albumin in the reaction volume would be between 0.07 and 0.1 mg·L^−1^, whereas the amount of CSF‐derived albumin is between 3 and 5 mg·L^−1^, so that the additional blood‐derived albumin should not play a major role for the seeding reaction. As the samples are incubated under comparably unphysiological conditions (pH 6.9, 50 °C and vigorous shaking), the blood cells are presumably quickly damaged leading to release of intracellular components or membrane fragments, which may contribute to the altered aggregation behaviour. Finally, it is very likely that not only one single factor, but several factors together contribute to the prolonged lag times observed. Based on our observations, we would recommend to carefully interpret the qRT‐QuIC results of CSF samples with visible contamination with blood in terms of quantitative aspects.

In this study, we show for the first time that the qRT‐QuIC seeding reaction is altered – particularly regarding quantitative aspects – under conditions, which are relevant in handling of clinical samples. The strength of our model with artificial seeds is that we can systematically and easily vary different parameters like freeze–thaw cycles and blood contamination and analyse their effects at different and defined amounts of seeds. However, one must be cautious with transferring the results one‐to‐one to real patient‐derived CSF samples. Nevertheless, the results of this study address an important aspect and represent a reasonable and solid basis for further considerations and investigations with real patient‐derived CSF specimens.

## Conclusions

With regard to clinical practicability and handling of CSF samples, we have used our model system and systematically characterized parameters which potentially interfere with the seeding reaction and thus lead to false‐positive or false‐negative results. Altogether, qRT‐QuIC turned out to be quite a robust assay in terms of sensitivity and specificity although quantitative interpretation, for example, in terms of disease progression or therapy monitoring might be impaired under certain conditions. Thus, explicit requirements to the quality of CSF samples would need to be defined. Although our findings still need to be confirmed using patient‐derived CSF samples, they provide a good starting point for further investigations.

## Conflict of interest

The authors declare no conflict of interest.

## Author contribution

AG and BM oversaw the project. VCR, SS, FS, DW, GSN and AG designed, performed, analysed and interpreted the experiments. BM and UK provided CSF samples. VCR wrote the manuscript. All authors critically read, edited and approved the manuscript.

## Supporting information


**Fig. S1.** Higher temperatures decrease apparent lag times. Compared to 50 °C (Fig. 2A), the seeding reaction is much slower at 37 °C.

**Fig. S2.** Different pre‐treatments of the seed preparation have no effect on their seeding activity. Ultracentrifugation (A) or sonication (B) of the seed preparation does not alter the seeding activity of the seed preparation. Error bars indicate standard deviation; *n* = 1.

**Fig. S3.** Multiple freeze–thaw cycles reduce ThT fluorescence of seeds diluted in ddH_2_O but not of seeds diluted in CSF. Seeds diluted in either ddH_2_O or CSF were subjected to 0, 1, or 5 freeze–thaw cycles before being added to the seeding reaction. Diagrams depict the baseline fluorescence at the beginning of the seeding reaction at a seed concentration of 500 nm, where a clear decrease of the ThT fluorescence is observed for seeds diluted in ddH_2_O but only a slight reduction of ThT fluorescence can be seen after five freeze–thaw cycles. Error bars indicate standard error of means; *n* = 3.
