# Supplementary figures and images for: Potential sources of interference with the highly sensitive detection and quantification of alpha‐synuclein seeds by qRT‐QuIC
Source: FEBS Open Bio. 2020 Apr 10;10(5):883–93. doi: 10.1002/2211-5463.12844 (PMC7193167; doi:10.1002/2211-5463.12844)

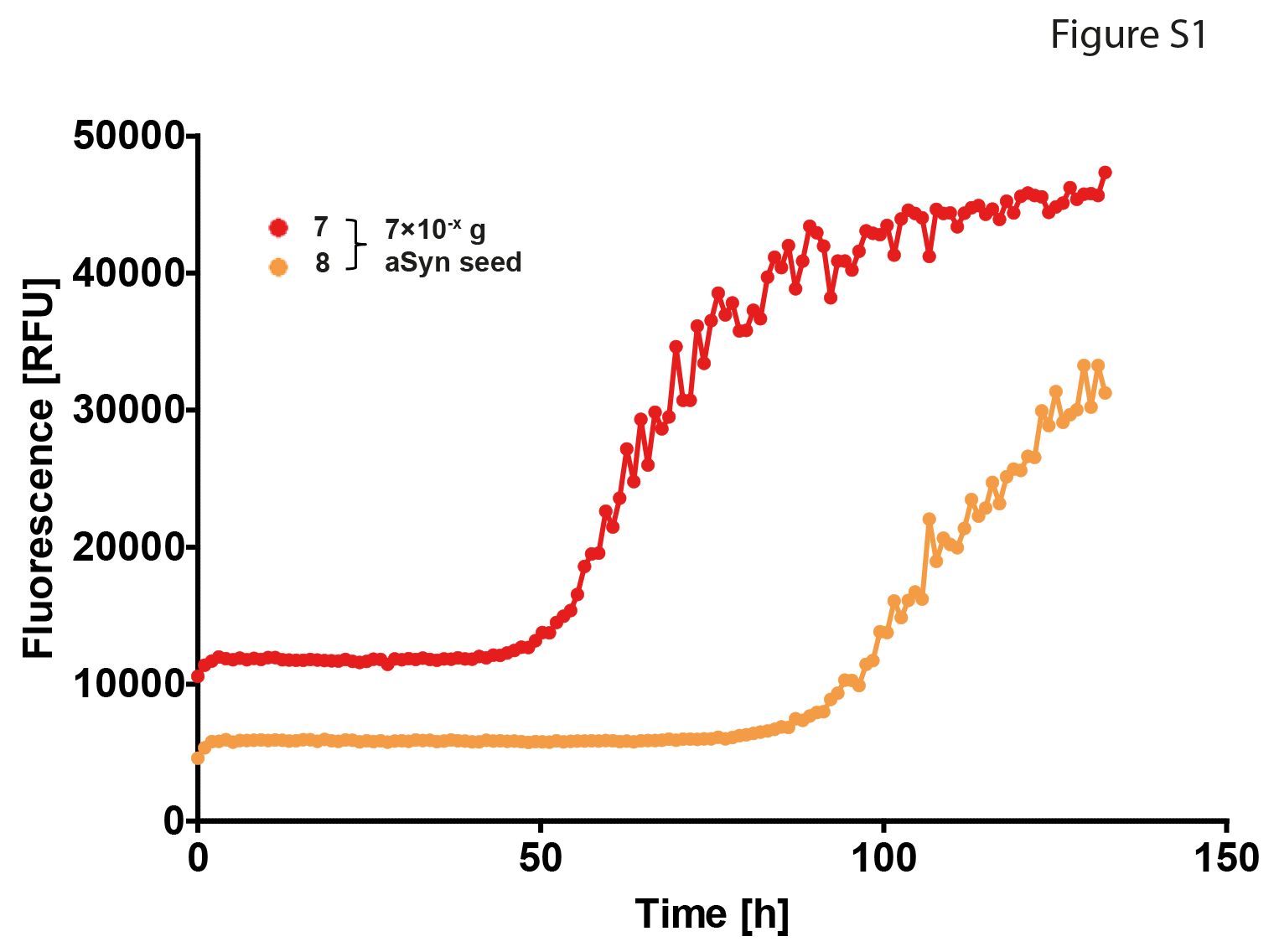

Supplement: Supplementary file 1 — Fig. S1. Higher temperatures decrease apparent lag times. Compared to 50 °C (Fig. 2A), the seeding reaction is much slower at 37 °C. [file FEB4-10-883-s001.tif]

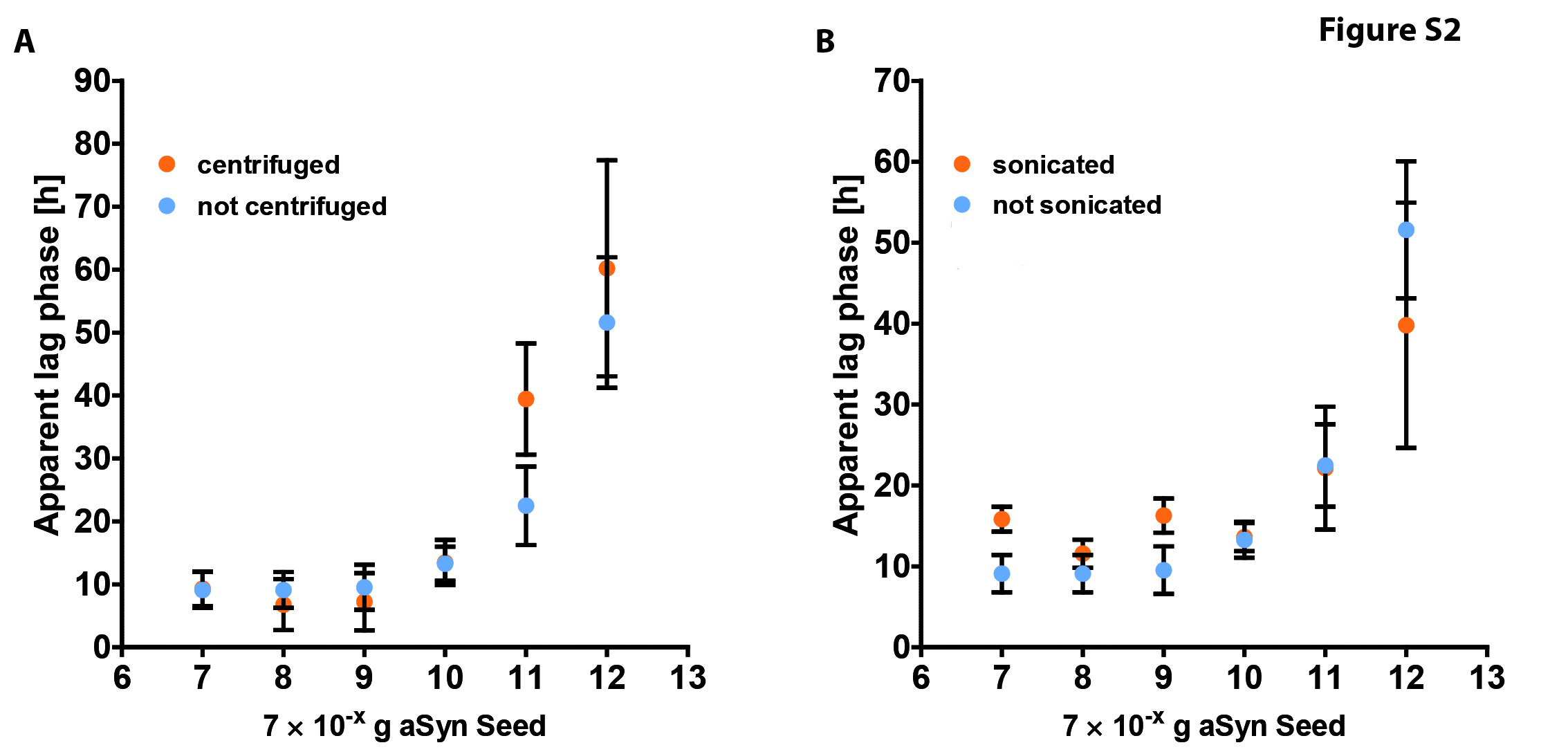

Supplement: Supplementary file 2 — Fig. S2. Different pre‐treatments of the seed preparation have no effect on their seeding activity. Ultracentrifugation (A) or sonication (B) of the seed preparation does not alter the seeding activity of the seed preparation. Error bars indicate standard deviation; n = 1. [file FEB4-10-883-s002.tif]

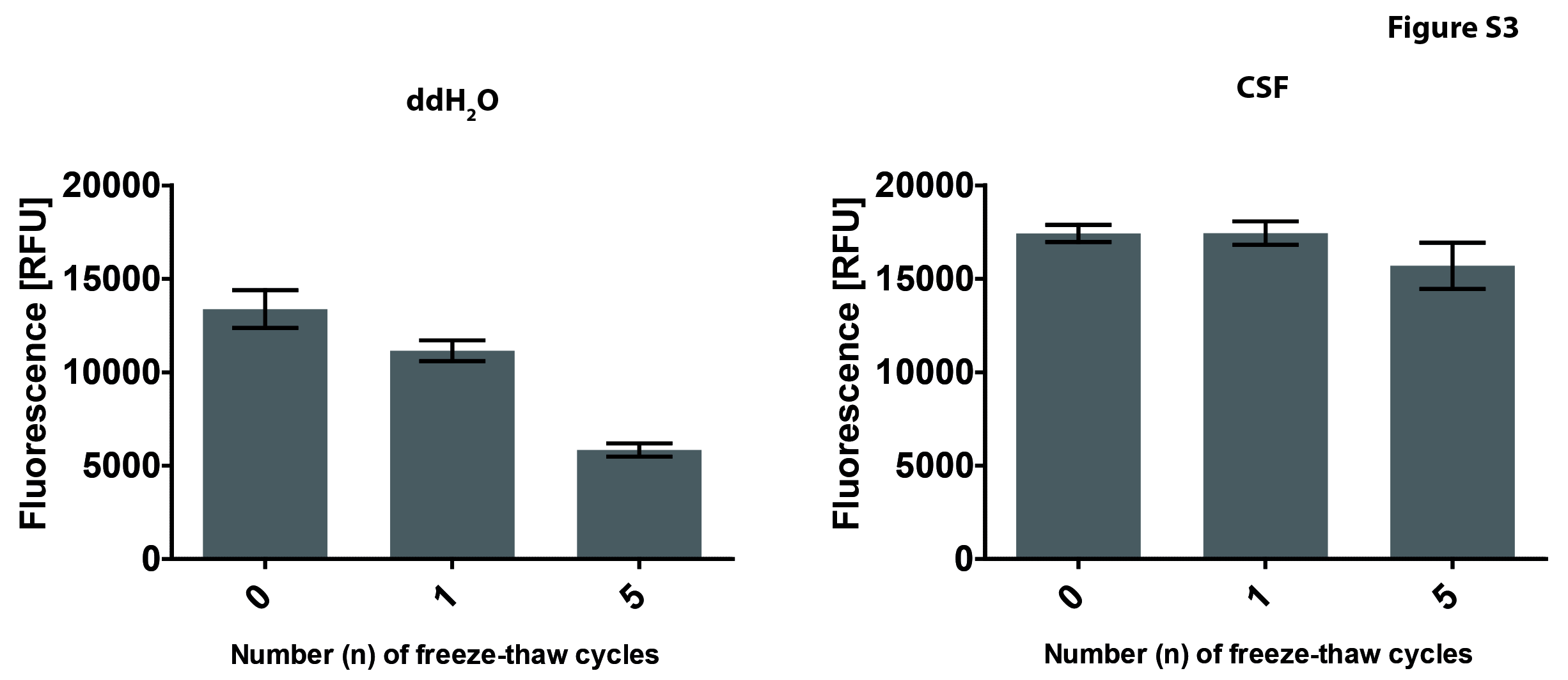

Supplement: Supplementary file 3 — Fig. S3. Multiple freeze–thaw cycles reduce ThT fluorescence of seeds diluted in ddH2O but not of seeds diluted in CSF. Seeds diluted in either ddH2O or CSF were subjected to 0, 1, or 5 freeze–thaw cycles before being added to the seeding reaction. Diagrams depict the baseline fluorescence at the beginning of the seeding reaction at a seed concentration of 500 nm, where a clear decrease of the ThT fluorescence is observed for seeds diluted in ddH2O but only a slight reduction of ThT fluorescence can be seen after five freeze–thaw cycles. Error bars indicate standard error of means; n = 3. [file FEB4-10-883-s003.tif]
